# Retraction: A significant causal association between C-reactive protein levels and schizophrenia

**DOI:** 10.1038/srep46947

**Published:** 2018-02-21

**Authors:** Masatoshi Inoshita, Shusuke Numata, Atsushi Tajima, Makoto Kinoshita, Hidehiro Umehara, Masahito Nakataki, Masashi Ikeda, Souichiro Maruyama, Hidenaga Yamamori, Tetsufumi Kanazawa, Shinji Shimodera, Ryota Hashimoto, Issei Imoto, Hiroshi Yoneda, Nakao Iwata, Tetsuro Ohmori

Correction to: Scientific Reports 6: Article number: 2610510.1038/srep26105; published online: 05 19 2016; updated: 02 21 2018

The authors are retracting this Article. The Article comprises three parts: a case-control study; a meta-analysis of case-control studies; and Mendelian randomization (MR) analyses (two datasets; a Japanese dataset and a public genome-wide association study [GWAS]-pooled dataset). In the MR analysis using the public dataset, the authors mistook effect alleles, resulting in incorrect results (Figure 3) and conclusion.

Our updated MR results are as follows: odds ratio (OR)scz/crp 0.87 (95% CI 0.76–1.00, p = 0.046) using rs2794520; 0.83 (95% CI 0.72–0.96, p = 0.014) using rs1183910; 0.85 (95% CI 0.77–0.94, p = 0.0017) across these two single nucleotide polymorphisms (SNPs); and 0.89 (95% CI 0.82–0.97, p = 0.006) using 15 C-reactive protein (CRP)-associated SNPs, which reached genome-wide significance in a meta-analysis of GWAS, respectively. These updated results of the MR analysis using the limited number of SNPs indicate a protective causal association between elevated CRP levels and schizophrenia risk. Therefore, the hypothesis in the conclusion of the Article—that medications which reduce CRP levels can be used in schizophrenia—is not supported.

All authors recognize these miscalculations in the original MR analysis and have agreed to the retraction of this Article.

